# Supplementary material for: Segmentation stability of human head and neck cancer medical images for radiotherapy applications under de-identification conditions: Benchmarking data sharing and artificial intelligence use-cases
Source: Front Oncol. 2023 Feb 28;13:1120392. doi: 10.3389/fonc.2023.1120392 (PMC10011442; doi:10.3389/fonc.2023.1120392)
Supplement: Supplementary file 1 [file DataSheet_1.pdf]

# Appendix A: Supplementary Data

For completeness, segmentation experiments were also quantified using additional surface distance metrics. These metrics were the mean Hausdorff distance at 95% ($MHD_{95}$) and the Hausdorff distance at 95% (95HD):

$$MHD_{95} = \frac{1}{2}(max_{P_{95}}\{d(t,P) \mid t \in T\} + max_{P_{95}}\{d(p,T)) \mid p \in P\}),$$
$$95HD = max\{max_{P_{95}}\{d(t,P) \mid t \in T\}, max_{P_{95}}\{d(p,T) \mid p \in P\}\},$$

where *P* the set of segmentation surface voxels of the model output, and *T* the set of segmentation surface voxels of the annotation. The distance from the surface metric is defined as: $d(a,B) = min_{b \in B}\{||a - b||_2\}$.

Additional metrics for the model training and model testing experiments are shown in **Figure A1** and **Figure A2**, respectively.

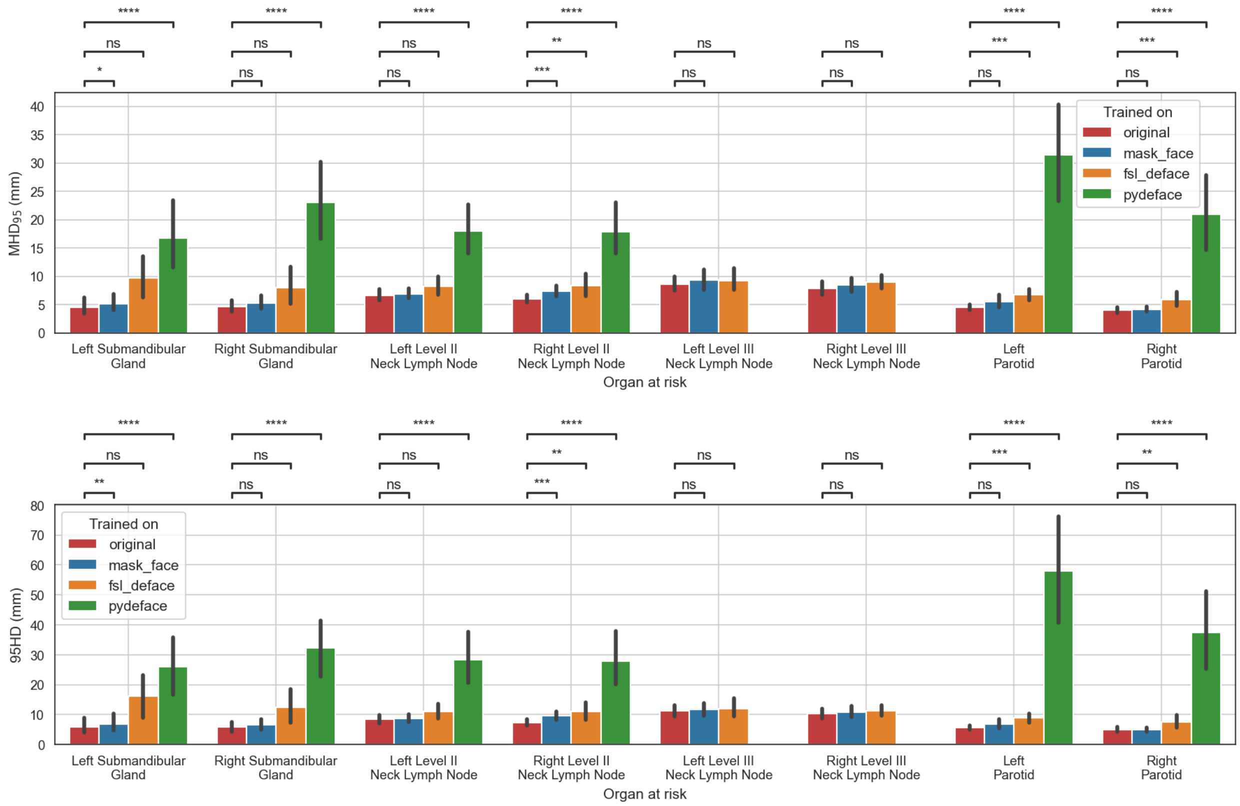


**Figure A1**. Additional surface metric values for performance of the models trained on original or defaced data and evaluated on the original data. The mean and standard deviation for each metric are represented as the center and extremes of the error bars, respectively. Statistical significance was determined using Wilcoxon signed-rank tests corrected with Benjamini-Hochberg procedure for all OARs and models. Comparison symbols: ns ($p > 0.05$), * ($p \leq 0.05$), ** ($p \leq 0.01$), *** ($p \leq$ 1e-4), **** ($p \leq$ 1e-5).

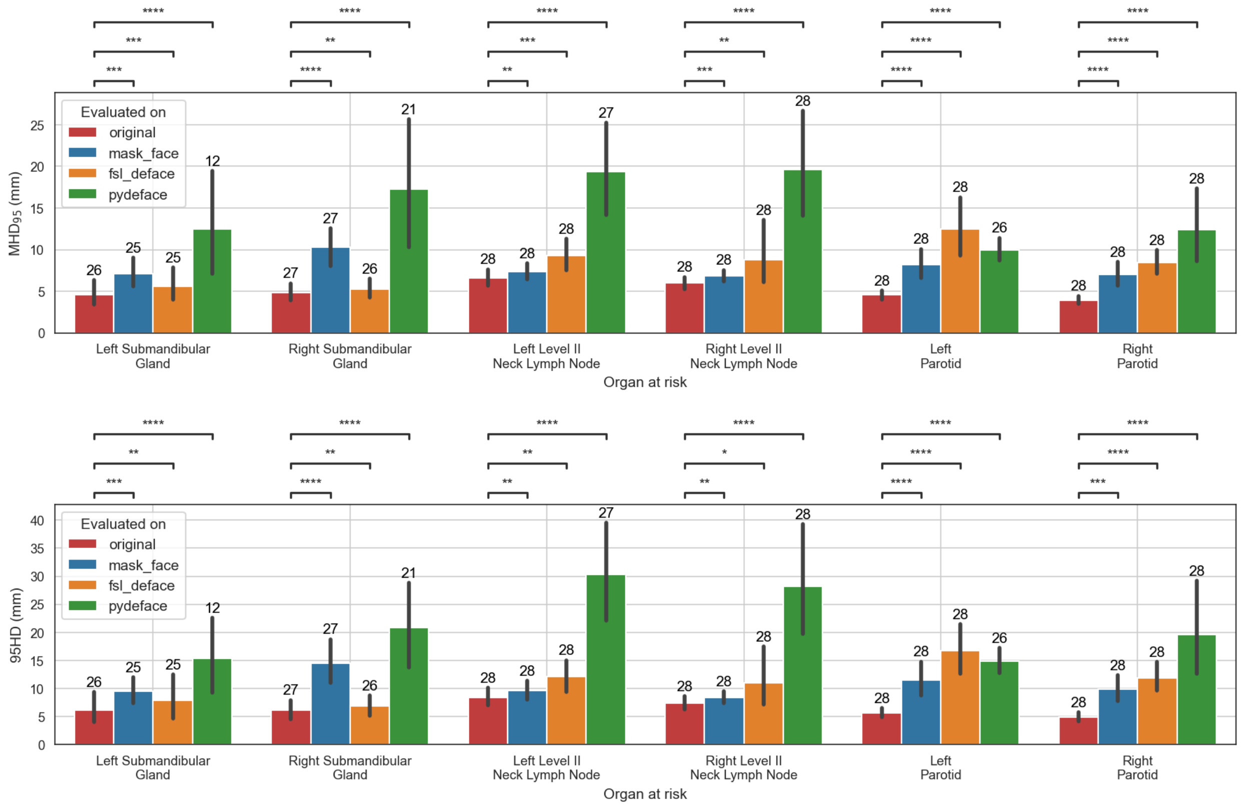


**Figure A2**. Additional surface metric values for performance of models trained on the original data when evaluated on the original, mask_face, fsl_deface, or pydeface data for the six organs at risk included in the analysis. Only cases that were available for all methods were included: 28 segmentations were used for all structures except in the case of the left and right submandibular glands where 26 and 27 segmentations were used, respectively. Empty model output segmentations were discarded, which resulted in a smaller number of evaluated structures. The number of evaluated structures is shown on top of the barplot. The mean and standard deviation for each metric are represented as the center and extremes of the error bars, respectively. Statistical significance was measured with Wilcoxon signed-rank tests corrected with Benjamini-Hochberg procedure for all OARs and models. Comparison symbols: ns ($p > $ Comparison symbols: ns ($p > 0.05$), * ($p \leq 0.05$), ** ($p \leq 0.01$), *** ($p \leq$ 1e-4), **** ($p \leq$ 1e-5).
